# Gene Therapy for β-Haemoglobinopathies: From Molecular Correction to Curative Medicine

**DOI:** 10.3390/biomedicines14071451

**Published:** 2026-06-26

**Authors:** Federica Fogliazza, Giulia Carbone, Martina Berzieri, Davide Ciriaco, Susanna Esposito

**Affiliations:** Pediatric Clinic, University Hospital of Parma, 43126 Parma, Italy; federica.fogliazza95@gmail.com (F.F.); giuucarbone@gmail.com (G.C.); martinaberzieri@gmail.com (M.B.); davide.ciriaco@unipr.it (D.C.)

**Keywords:** β-haemoglobinopathies, sickle cell disease, transfusion-dependent β-thalassaemia, gene therapy, gene addition, gene editing

## Abstract

**Background:** β-haemoglobinopathies, including sickle cell disease and transfusion-dependent β-thalassaemia, are among the most common monogenic disorders worldwide and represent a major global health burden. Conventional treatments, such as blood transfusions, iron chelation, fetal haemoglobin induction, and allogeneic haematopoietic stem cell transplantation, have improved outcomes but remain limited by treatment-related toxicity, donor availability, and incomplete curative potential. **Methods:** A narrative literature review was conducted using PubMed up to 2025. Search terms included “sickle cell disease,” “sickle cell anemia,” “β-thalassemia,” “transfusion-dependent beta-thalassemia,” “gene therapy,” “gene addition,” “gene editing,” “CRISPR-Cas9,” “lentiviral vector,” “children,” “paediatric,” and “pediatric.” Relevant clinical trials, reviews, consensus statements, and guidelines were selected and qualitatively analysed. **Results:** Gene therapy for β-haemoglobinopathies is based mainly on two strategies: gene addition and gene editing. Gene addition uses lentiviral vectors to introduce functional or modified β-globin genes into autologous haematopoietic stem cells, whereas gene editing targets regulatory pathways, particularly *BCL11A*, to reactivate fetal haemoglobin synthesis or correct disease-causing mutations. Clinical studies have shown encouraging outcomes, including transfusion independence in many patients with β-thalassaemia and marked reduction or elimination of vaso-occlusive crises in sickle cell disease. Paediatric and adolescent data are increasingly promising, although still limited. **Conclusions:** Gene therapy is reshaping the treatment landscape of β-haemoglobinopathies by offering a personalised and potentially curative approach. However, long-term safety, conditioning toxicity, fertility preservation, accessibility, costs, and implementation in high-prevalence regions remain critical challenges. Further studies are needed to optimise patient selection and expand equitable access.

## 1. Introduction

Haemoglobinopathies are the most common monogenic disorders worldwide. Approximately 7% of the global population carries a DNA variant affecting haemoglobin synthesis, giving rise to a heterogeneous group of conditions that include α- and β-thalassaemias, sickle cell disease, and haemoglobin variants associated with altered erythropoiesis [1,2,3,4].

Among these disorders, transfusion-dependent β-thalassaemia (TDT) and sickle cell disease (SCD) represent the greatest clinical and public health burden. Each year, an estimated 60,000 new cases of TDT and 300,000 new cases of SCD are diagnosed worldwide [5]. Both conditions result from alterations affecting the β-globin gene, which encodes the β chains of adult haemoglobin [5].

In TDT, more than 300 pathogenic variants have been described. These mutations lead to either reduced (β^+^) or absent (β^0^) β-globin chain production, resulting in an imbalance between α- and β-globin chains. The consequent accumulation of unmatched α chains impairs erythroid maturation, causes ineffective erythropoiesis, and leads to chronic anaemia requiring regular transfusion support [6].

In SCD, a single nucleotide substitution in the β-globin gene causes the replacement of glutamic acid with valine, producing sickle haemoglobin. Under conditions such as hypoxia, dehydration, or physiological stress, sickle haemoglobin polymerises, deforming red blood cells into the characteristic sickle shape. This process promotes haemolysis, recurrent vaso-occlusive events, chronic inflammation, progressive organ damage, and reduced life expectancy [7,8,9].

Given their high prevalence, lifelong morbidity, and impact on survival and quality of life, haemoglobinopathies represent a major and growing global health challenge [3]. Conventional management has historically relied on supportive approaches, including regular blood transfusions, iron chelation therapy, and pharmacological induction of fetal haemoglobin synthesis [4]. Allogeneic haematopoietic stem cell transplantation (allo-HSCT) has long represented the only established curative option. However, its use is limited by the availability of suitable donors, the risk of graft rejection and graft-versus-host disease, conditioning-related toxicity, and significant treatment-associated morbidity and mortality [4,10,11].

A deeper understanding of haemoglobin biology, erythropoiesis, and the molecular mechanisms underlying β-haemoglobinopathies has enabled the development of gene-based therapeutic strategies. These approaches use autologous haematopoietic stem cells and aim either to introduce a functional β-globin gene or to modify endogenous regulatory pathways, particularly those controlling fetal haemoglobin expression. As a result, autologous gene therapy is emerging as a potentially curative strategy that may complement, and in selected cases provide an alternative to, allogeneic transplantation [12].

The aim of this narrative review is to provide an overview of the current gene therapy landscape for β-haemoglobinopathies, with a focus on SCD and TDT. In particular, this review describes the main technologies currently under investigation, highlights the key differences between gene addition and gene editing approaches, and discusses available clinical outcomes and safety considerations. Unlike recent reviews that mainly address either the molecular basis of gene transfer and genome editing or the efficacy of individual products, this review specifically integrates gene addition and gene-editing approaches for both SCD and TDT, with particular emphasis on paediatric and adolescent applicability, clinical pathway differences, patient-selection criteria, safety considerations, and implementation challenges in routine care.

## 2. Methods

A comprehensive literature search was conducted in PubMed to identify relevant articles published up to 2025. The search strategy included combinations of the following keywords and Medical Subject Headings, where applicable: “sickle cell disease,” “sickle cell anemia,” “β-thalassemia,” “beta-thalassemia,” “transfusion-dependent beta-thalassemia,” “hemoglobinopathies,” “gene therapy,” “gene addition,” “gene editing,” “genome editing,” “CRISPR-Cas9,” “lentiviral vector,” “children,” “paediatric,” and “pediatric.”.

Boolean operators were used to combine search terms, including: “sickle cell disease” OR “sickle cell anemia”; “beta-thalassemia” OR “transfusion-dependent beta-thalassemia”; “gene therapy” OR “gene addition” OR “gene editing”; and “children” OR “paediatric” OR “pediatric.” Additional relevant publications were identified by screening the reference lists of selected articles and key reviews.

The search focused on original clinical studies, clinical trials, review articles, consensus statements, and guidelines addressing gene therapy approaches for β-haemoglobinopathies, particularly sickle cell disease and transfusion-dependent β-thalassaemia. Studies were considered relevant if they described gene addition or gene editing strategies, clinical outcomes, safety data, patient eligibility criteria, or paediatric applications. Articles not written in English, studies not focused on haemoglobinopathies, and publications without direct relevance to gene therapy were excluded.

Given the narrative nature of this review, no formal systematic review protocol was registered, and no quantitative meta-analysis was performed. The selected literature was analysed qualitatively, with particular attention to therapeutic mechanisms, available clinical evidence, safety considerations, and current limitations of gene therapy in both adult and paediatric populations.

## 3. Gene Therapy

Haemoglobin synthesis is a tightly regulated developmental process controlled by the α-globin gene cluster on chromosome 16 and the β-globin gene cluster on chromosome 11. During fetal life, erythropoiesis is characterised by predominant production of fetal haemoglobin (HbF), composed of two α-globin and two γ-globin chains. After birth, a physiological “haemoglobin switch” occurs, usually within the first months of life, leading to progressive silencing of γ-globin expression and replacement of HbF by adult haemoglobin (HbA). This process is largely regulated by transcriptional repressors, among which *BCL11A* plays a central role in inhibiting γ-globin expression [3].

The clinical manifestations of β-haemoglobinopathies generally become evident after this switch, when defective or abnormal β-globin production becomes functionally dominant [13,14,15]. In both β-thalassaemia and SCD, persistence of HbF has a protective effect. Hereditary persistence of fetal haemoglobin (HPFH), a naturally occurring condition characterised by continued γ-globin expression into adulthood, can reduce disease severity and, in some cases, markedly attenuate or prevent clinical manifestations [16]. This observation has provided a strong biological rationale for therapeutic strategies aimed at either restoring functional β-globin production or reactivating endogenous HbF synthesis.

Advances in molecular biology, viral vector engineering, and genome-editing technologies have made gene therapy a promising curative approach for β-haemoglobinopathies. Current strategies are broadly divided into two main categories: gene addition, which introduces a functional or modified β-globin gene into autologous haematopoietic stem and progenitor cells, and gene editing, which directly modifies genomic sequences involved in haemoglobin regulation or disease pathogenesis.

### 3.1. Gene Addition

Gene addition is based on the *ex vivo* modification of autologous CD34+ haematopoietic stem and progenitor cells through the insertion of a functional β-globin or β-globin–like therapeutic cassette. This approach has evolved over the past two decades through the development of increasingly efficient and safer viral vectors, together with the incorporation of key regulatory elements from the β-globin locus control region, particularly hypersensitive sites, which enhance erythroid-specific and sustained transgene expression [1,17,18].

Several viral vector platforms have been explored for gene transfer, including adenoviral, adeno-associated viral, retroviral, and lentiviral vectors. Among these, lentiviral vectors have become the preferred platform for β-haemoglobinopathies because they can efficiently transduce both dividing and non-dividing haematopoietic stem cells and allow stable integration of the therapeutic cassette into the host genome [1,19,20]. In current clinical applications, replication-incompetent lentiviral vectors are designed to deliver the therapeutic gene while minimising the risk of generating replication-competent virus. Their ability to engineer autologous CD34+ cells enables long-term production of corrected erythroid progeny after myeloablative conditioning and reinfusion of the modified cells [21].

In β-thalassaemia, gene addition aims to restore β-globin synthesis, reduce the α/non-α globin chain imbalance, improve ineffective erythropoiesis, and ultimately achieve transfusion independence. In SCD, the therapeutic objective is to introduce an anti-sickling β-globin variant capable of reducing the relative proportion and polymerisation of sickle haemoglobin, thereby limiting haemolysis and vaso-occlusive complications.

### 3.2. Gene Editing

Gene editing represents a distinct therapeutic strategy based on the targeted modification of specific genomic regions. In β-haemoglobinopathies, most current approaches aim to reactivate HbF synthesis by disrupting regulatory elements that normally repress γ-globin expression. In β-thalassaemia, increased HbF can compensate for deficient β-globin chain production, whereas in SCD it reduces the intracellular concentration of sickle haemoglobin and inhibits polymerisation, thereby preventing erythrocyte sickling [5,22,23,24].

This strategy reproduces, in therapeutic form, the protective effect observed in HPFH. Genome-editing platforms such as zinc-finger nucleases, transcription activator-like effector nucleases, and CRISPR-Cas systems can introduce targeted DNA modifications that alter regulatory sequences or silence repressors of γ-globin expression [11,25]. Among these targets, the erythroid-specific enhancer of *BCL11A* has emerged as one of the most clinically relevant, because its disruption reduces *BCL11A* expression selectively in erythroid cells while preserving its functions in other lineages.

Clinical studies by Frangoul et al. and Locatelli et al. have demonstrated the therapeutic potential of this approach using exagamglogene autotemcel (exa-cel), an autologous CD34+ cell product edited ex vivo with CRISPR-Cas9 to disrupt the erythroid-specific enhancer of *BCL11A* [5,26,27]. By removing the transcriptional repression of γ-globin, exa-cel induces robust and broadly distributed HbF production, with fetal haemoglobin levels exceeding 20% in treated patients and translating into sustained clinical benefit. In patients with severe SCD, this benefit has been reflected by prolonged freedom from vaso-occlusive crises, whereas in patients with TDT, including those with severe β^0^/β^0^ genotypes, treatment has resulted in transfusion independence or marked reduction in transfusion requirements [26,27].

Clinical benefit in these studies was assessed using disease-specific endpoints, including sustained absence of vaso-occlusive crises in SCD, transfusion independence in TDT, increases in total haemoglobin and HbF levels, improvement in haemolysis and iron-overload markers, discontinuation of iron chelation when appropriate, and improvements in patient-reported quality of life [26,27]. Table 1 summarizes the main clinical outcomes used to assess the efficacy of gene therapy in SCD and TDT.

## 4. Gene Therapy for Paediatric Sickle Cell Disease

Both gene addition and gene editing strategies have been investigated for the treatment of paediatric SCD. These approaches are based on the ex vivo genetic modification of autologous haematopoietic stem and progenitor cells, usually CD34+ cells, followed by reinfusion after myeloablative conditioning. Gene addition relies on lentiviral vectors to introduce a therapeutic β-globin gene, whereas gene editing uses programmable nucleases, most commonly CRISPR-Cas systems, to modify genomic regions involved in SCD pathogenesis or haemoglobin regulation [28].

In gene addition approaches, lentiviral vectors may be used either to insert a modified β-globin gene capable of producing an anti-sickling haemoglobin variant, such as HbA^T87Q, or to deliver regulatory sequences, including short hairpin RNA (shRNA), designed to downregulate *BCL11A* and thereby increase γ-globin expression and HbF production [28,29]. In contrast, gene editing strategies initially focused on disrupting the erythroid-specific *GATA1*-binding region within the *BCL11A* enhancer, with the aim of reactivating γ-globin synthesis and increasing HbF levels [23,27,28,30,31,32,33].

More recently, newer genome-editing platforms have been developed to improve precision and reduce the potential risks associated with double-stranded DNA breaks. These include cytosine and adenine base editors, which can introduce targeted nucleotide substitutions without inducing conventional double-strand breaks. Through these approaches, either the pathogenic SCD mutation itself or regulatory sequences within the *BCL11A* erythroid-specific enhancer can be targeted, leading, respectively, to restoration of normal HbA production or increased HbF expression [28,29,34,35,36,37,38,39]. Additional strategies using CRISPR-Cas12 systems to target γ-globin promoter regions have also shown promising preclinical results [28,40].

At present, two autologous haematopoietic stem cell gene therapies have been approved by the U.S. Food and Drug Administration (FDA) for patients with SCD aged 12 years and older and with a history of recurrent vaso-occlusive crises (VOCs). In December 2023, the FDA approved lovotibeglogene autotemcel, also known as lovo-cel or Lyfgenia^®^ (bluebird bio, Inc., Somerville, MA, USA), and exagamglogene autotemcel, also known as exa-cel or Casgevy^®^ (Vertex Pharmaceuticals Incorporated, Boston, MA, USA) [26,41,42,43]. Lovo-cel is a one-time ex vivo lentiviral gene addition therapy that introduces a modified β-globin gene into autologous CD34+ cells, resulting in production of the anti-sickling haemoglobin HbA^T87Q [41,42,43]. Exa-cel is a one-time ex vivo CRISPR-Cas9-based gene editing therapy that disrupts the erythroid-specific enhancer of *BCL11A*, thereby reactivating endogenous HbF production [26]. Thus, both a gene addition strategy and a gene editing strategy are currently available for selected adolescents and adults with SCD. Criteria for patient selection and for choosing between these approaches are discussed below.

The clinical pathway for SCD gene therapy includes several sequential phases: baseline evaluation, transfusion optimisation, stem cell mobilization, apheresis, product manufacturing, myeloablative conditioning, infusion of the gene-modified cellular product, and post-infusion monitoring. After a complete clinical assessment, patients generally initiate or continue a transfusion programme for approximately 8–12 weeks before stem cell collection. The objective is to reduce sickle haemoglobin to below 30% and maintain total haemoglobin between approximately 9 and 11 g/dL, thereby decreasing the risk of VOCs and other SCD-related complications during mobilisation and apheresis [28,44].

Mobilisation is usually performed only when the patient has been free from VOCs for at least 7–14 days. In SCD, granulocyte colony-stimulating factor (G-CSF) is contraindicated because of the risk of hyperviscosity, inflammation, and VOCs. Therefore, mobilization is performed using plerixafor, usually administered 2–4 h before apheresis [28,44,45,46]. The target cell dose is substantially higher than that required for conventional autologous transplantation, generally around 15–20 × 10^6^ CD34+ cells/kg, because cell loss may occur during ex vivo manufacturing and quality-control procedures [28,44,45,46]. Consequently, some patients require multiple mobilisation and apheresis cycles, and in a minority of cases, inadequate collection may prevent treatment from proceeding.

Once sufficient CD34+ cells have been collected, the product is sent for manufacturing. This interval, which may last several weeks, can be used to continue transfusion support, complete pre-transplant reassessment, and address fertility preservation, which is particularly important in paediatric and adolescent patients because of the gonadotoxic effects of myeloablative conditioning [28,44]. When the manufactured product is available, and the patient remains eligible, busulfan-based myeloablative conditioning is administered, followed by infusion of the gene-modified autologous cells and careful short- and long-term monitoring.

### 4.1. Exa-Cel Versus Lovo-Cel

Exa-cel and lovo-cel differ substantially in their mechanisms of action. Lovo-cel is a gene addition therapy that enables erythroid cells to produce the modified anti-sickling haemoglobin HbA^T87Q, whereas exa-cel is a gene editing therapy that reactivates endogenous HbF production through disruption of the *BCL11A* erythroid enhancer [26,43,47,48]. Although these mechanisms are biologically distinct, both aim to reduce intracellular sickle haemoglobin polymerisation, prevent erythrocyte sickling, decrease haemolysis, and reduce the frequency of VOCs. The main characteristics of the two approved gene therapy products for SCD are summarized in Table 2.

Clinical data indicate that both therapies are highly effective in reducing severe VOCs. In the reported studies, approximately 93% of evaluable patients treated with lovo-cel achieved complete resolution of VOCs for a continuous period of at least 12 months, while approximately 97% of patients treated with exa-cel remained free from VOCs over a comparable assessment period [26,43,47,48]. Although these results appear numerically favourable for exa-cel, direct comparison should be interpreted with caution because the studies differed in design, eligibility criteria, baseline patient characteristics, and follow-up duration.

Notably, the lovo-cel studies included broader eligibility criteria than the exa-cel trials. Patients enrolled in lovo-cel protocols could include older individuals, patients with lower performance status, and, in some study phases, individuals with a history of overt stroke. In contrast, the exa-cel programme applied more restrictive criteria, including exclusion of some patients with chronic pain or specific prior complications [47]. Among lovo-cel-treated patients with previous overt stroke, available follow-up data showed maintenance of transfusion independence and no recurrent strokes over extended observation periods, although longer-term evidence remains necessary [47].

Differences also exist in the manufacturing process and cell collection requirements. Because CRISPR-Cas9 editing and subsequent product processing may be associated with additional haematopoietic stem cell loss, the recommended CD34+ cell collection target for exa-cel is higher than that for lovo-cel. The FDA label recommends a collection target of approximately 20 × 10^6^ CD34+ cells/kg for exa-cel compared with approximately 16.5 × 10^6^ CD34+ cells/kg for lovo-cel [47]. This distinction may be relevant in clinical practice, particularly for paediatric patients, patients with poor mobilisation, or those requiring repeated apheresis procedures.

Despite these differences, both therapies have shown similar overall biological and functional effects. They increase total haemoglobin, raise the proportion of anti-sickling haemoglobin, and lead to near-normalisation of haemolysis markers in many patients [26,43,47,48]. Clinically, episodes of severe acute pain requiring medical care are markedly reduced or eliminated. However, the interpretation of pain outcomes remains complex, particularly in patients with established chronic pain, central sensitisation, opioid exposure, or post-treatment pain syndromes [47].

The adverse events reported with both treatments have generally been consistent with the known toxicities of myeloablative busulfan conditioning and the underlying risks of SCD. These include cytopenias, infection risk, mucositis, febrile neutropenia, hepatic toxicity, and potential gonadal impairment. Long-term follow-up remains essential to assess the durability of efficacy, clonal dynamics, insertional mutagenesis risk for integrating vectors, off-target or unintended editing effects, and late treatment-related malignancies [26,43,47,48].

In paediatric and adolescent populations, both therapies have shown particularly encouraging results. In the lovo-cel programme, 14 paediatric patients were treated using the current manufacturing process, and all evaluable paediatric participants achieved complete resolution of VOCs during 6–18 months of post-infusion follow-up [43,47]. Importantly, patients with prior overt or silent stroke remained stroke-free and transfusion-independent during extended follow-up. Similarly, all evaluable paediatric participants treated with exa-cel achieved at least 12 consecutive months without VOCs [26,47]. Adolescents treated with exa-cel also reported improvements in general health, well-being, physical functioning, emotional and social functioning, and pain experience [49].

Overall, exa-cel and lovo-cel represent major advances in the treatment of severe SCD and provide clinically meaningful benefit in selected paediatric and adolescent patients. However, current evidence is derived mainly from non-randomised, open-label, single-arm studies. Differences in trial design, eligibility criteria, cell collection targets, manufacturing processes, and follow-up duration limit direct comparison between the two products [47,48]. Further real-world data and longer follow-up will be necessary to clarify comparative effectiveness, durability of response, long-term organ protection, fertility outcomes, safety, accessibility, and optimal patient selection.

Additional gene therapy strategies for paediatric SCD are currently under investigation, including new lentiviral gene addition products, CRISPR-Cas9-based approaches, base-editing platforms, and alternative nuclease systems. Ongoing clinical trials listed in ClinicalTrials.gov continue to expand the therapeutic landscape and may help define future indications for younger children and for patients with different disease severities or comorbidity profiles (Table 3) [50].

### 4.2. Inclusion Criteria

**Figure 1:**
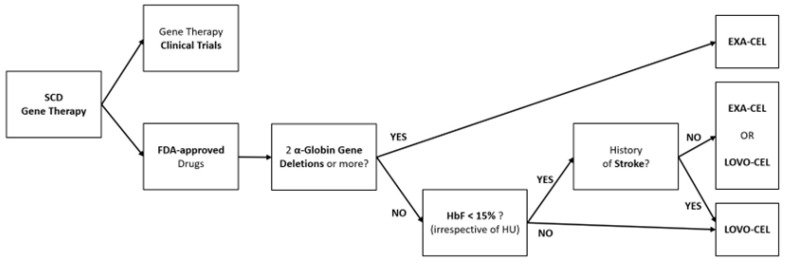
FDA-based decision pathway for selecting gene therapy in patients with sickle cell disease. Adapted from reference [28]. Abbreviations: FDA, U.S. Food and Drug Administration; SCD, sickle cell disease; lovo-cel, lovotibeglogene autotemcel; exa-cel, exagamglogene autotemcel; HSCs, haematopoietic stem cells; VOCs, vaso-occlusive crises.

Appropriate patient selection is essential to maximise the benefit–risk balance of gene therapy in sickle cell disease. Eligibility should be assessed through a multidisciplinary evaluation that considers disease severity, previous complications, response to standard therapies, organ function, transplant fitness, and the availability of alternative curative options, including an HLA-matched family donor. In this review, the main criteria proposed by the joint consensus conference of the European Hematology Association Specialized Working Group and the European Society for Blood and Marrow Transplantation Hemoglobinopathies Working Party are summarized in Table 4, while the FDA-based framework for selecting between currently approved gene therapy products is presented in Figure 1 [28,41].

## 5. Gene Therapy for Transfusion-Dependent β-Thalassaemia

As in SCD, gene therapy has emerged as a potentially curative therapeutic option for TDT. However, the biological rationale differs between the two disorders. In SCD, the main objective is to prevent haemoglobin S polymerisation and erythrocyte sickling, whereas in TDT the therapeutic goal is to restore effective β-like globin production, correct the imbalance between α- and β-globin chains, reduce the toxic accumulation of free α chains, and improve ineffective erythropoiesis [51,52].

Both gene addition and gene editing strategies are currently relevant for TDT. Gene addition aims to introduce a functional β-globin gene into autologous haematopoietic stem and progenitor cells, whereas gene editing aims mainly to reactivate HbF production by targeting regulators of γ-globin expression, such as the erythroid-specific enhancer of *BCL11A*. As in SCD, the therapeutic pathway includes baseline evaluation, stem cell mobilisation and collection, ex vivo cell manufacturing, myeloablative conditioning, infusion of the gene-modified cellular product, and long-term follow-up. Nevertheless, several disease-specific differences distinguish the clinical pathway in TDT from that used in SCD, particularly with regard to mobilisation strategy, transfusion optimisation, iron overload assessment, and post-treatment management [28,44].

### 5.1. Clinical Pathway for TDT Gene Therapy

#### 5.1.1. Phase A: Eligibility Assessment and Fertility Preservation

The first step is the careful identification of patients who may benefit from gene therapy and who are sufficiently fit to tolerate the procedure. In 2021, the Italian Society of Thalassaemias and Haemoglobinopathies (SITE) developed a consensus statement for selecting patients with β-thalassaemia for gene therapy, which was subsequently considered within the broader European haematology context [53]. According to this framework, eligibility should be based on transfusion burden, age, disease severity, organ function, comorbidities, and the patient’s ability to undergo myeloablative conditioning.

Transfusion dependence is a key criterion and is generally defined as a transfusion-free interval of no more than 6 weeks during the previous two years, or an annual transfusion requirement of at least 100 mL/kg of packed red blood cells over the same period [54]. Candidates should be followed regularly in a comprehensive haemoglobinopathy centre with expertise in transfusion management, iron chelation, organ monitoring, and transplant or cellular therapy procedures [55]. In most current frameworks, patients should be older than 12 years, while an upper age limit is generally interpreted in relation to organ function, comorbidities, and overall transplant fitness [53].

Because gene therapy currently requires busulfan-based myeloablative conditioning, a rigorous pre-treatment assessment is mandatory. Particular attention should be paid to iron-related organ damage. Patients with significant hepatic iron overload, commonly defined by liver iron concentration (LIC) greater than 7 mg Fe/g dry weight, may require intensified chelation before proceeding to gene therapy [53]. Cardiac evaluation is equally important: active myocardial iron overload, reflected by a cardiac T2* magnetic resonance imaging value below 10 ms, pulmonary hypertension, advanced heart failure, recent ischaemic events, restrictive cardiomyopathy, or clinically relevant arrhythmias may substantially increase procedural risk [53].

Hepatic status should also be carefully assessed, as advanced fibrosis or cirrhosis increases the risk of conditioning-related complications, including sinusoidal obstruction syndrome/veno-occlusive disease. Optimal candidates should have absent or minimal liver fibrosis, controlled iron overload, and no active viral hepatitis. Patients with occult or chronic hepatitis B virus infection require strict antiviral prophylaxis and specialist evaluation before treatment [53]. Additional comorbidities may influence eligibility. Diabetes mellitus may impair the bone marrow microenvironment and reduce stem cell mobilisation capacity, while renal dysfunction requires careful risk assessment. Hypersplenism may also negatively affect engraftment and should be considered during candidate selection [53,56].

On the basis of these criteria, patients may be classified as high-priority candidates, temporarily non-eligible patients requiring optimisation and re-evaluation, or permanently non-eligible patients because of excessive procedural risk [53]. However, eligibility criteria are evolving rapidly as clinical experience increases. Recent recommendations emphasise that final candidate selection should balance three elements: the intrinsic risk of the underlying disease, the expected risk of the gene therapy procedure, including conditioning toxicity, and the availability of appropriate expertise and gene therapy platforms at the treating centre [57].

Once eligibility has been established, fertility preservation should be addressed before conditioning. This is particularly important in children, adolescents, and young adults, as myeloablative busulfan is associated with a substantial risk of gonadal damage and permanent infertility [58].

#### 5.1.2. Phase B: Haematopoietic Stem Cell Mobilisation

Before mobilisation, patients with TDT should be clinically optimised. During the 8–12 weeks preceding stem cell collection, transfusion support is generally intensified to maintain haemoglobin levels around or above 11 g/dL, with the aim of improving patient condition and supporting an adequate stem cell harvest [44].

Unlike in SCD, granulocyte colony-stimulating factor (G-CSF) can be safely used in TDT. Therefore, the combination of G-CSF and plerixafor is considered the preferred mobilisation strategy, as it allows efficient and relatively rapid collection of CD34+ haematopoietic stem and progenitor cells [59]. This represents a major difference from SCD, where G-CSF is avoided because of the risk of vaso-occlusive complications.

#### 5.1.3. Phase C: Apheresis and Cellular Manufacturing

After successful mobilisation, CD34+ cells are collected by apheresis. Because a proportion of cells may be lost during ex vivo genetic modification, quality-control procedures, cryopreservation, and product release, the target collection dose is higher than that required for conventional autologous transplantation. A target yield of approximately 15–20 × 10^6^ CD34+ cells/kg is generally recommended to allow manufacturing of the therapeutic product while preserving an adequate back-up graft if needed [44].

The collected cells are then transferred to specialised manufacturing facilities, where they undergo either lentiviral transduction for gene addition or genome editing, depending on the therapeutic platform used. During this manufacturing interval, patients continue standard supportive care, including transfusion and chelation therapy, and undergo repeat clinical assessment to confirm ongoing eligibility [44].

#### 5.1.4. Phase D: Myeloablative Conditioning

Before infusion of the gene-modified product, patients receive myeloablative conditioning, most commonly with busulfan. Busulfan depletes endogenous haematopoietic stem cells and creates marrow niches that allow engraftment of the modified autologous cells [60]. Because busulfan has a narrow therapeutic window and relevant interpatient pharmacokinetic variability, careful dosing, therapeutic drug monitoring, and organ toxicity surveillance are essential, particularly in paediatric patients and in individuals with pre-existing iron-related organ damage [44,60].

#### 5.1.5. Phase E: Reinfusion and Early Post-Transplant Supportive Care

After completion of conditioning and adequate busulfan washout, usually within 2–7 days, the gene-modified cellular product is thawed and infused intravenously [44]. Early post-infusion management is similar to that used after autologous haematopoietic stem cell transplantation and includes monitoring for neutrophil and platelet recovery, transfusion support, infection prophylaxis, mucositis management, and surveillance for hepatic, renal, and other conditioning-related toxicities.

#### 5.1.6. Phase F: Long-Term Follow-Up

Long-term follow-up is essential to evaluate durability, safety, and late effects. Because myeloablative conditioning causes profound immunosuppression, patients require antimicrobial prophylaxis during the early post-transplant period, including antibacterial, antiviral, and antifungal measures according to institutional protocols [44].

Transfusion independence is typically achieved within 3–6 months after infusion in responding patients. Once transfusions are discontinued and erythropoiesis stabilises, management of residual iron overload becomes a major objective. In patients with adequate haemoglobin levels, therapeutic phlebotomy is often preferred over continued chelation to remove excess iron stores [44].

Long-term surveillance should include regular assessment of haemoglobin levels, vector copy number or editing durability according to the product used, clonal haematopoiesis, organ function, iron burden, endocrine function, and fertility-related outcomes. Gynaecological, andrological, and endocrinological evaluations are particularly important in children, adolescents, and young adults because of the risk of gonadal failure after busulfan conditioning. Follow-up visits should continue at progressively extended intervals to monitor the stability of engraftment, durability of clinical benefit, and potential late adverse events [44].

### 5.2. Genotype-Dependent Responses and Evolving Genetic Strategies

β-thalassaemia is characterised by a highly heterogeneous mutational landscape, and the underlying genotype can influence response to gene therapy. In the early development of lentiviral gene addition, genotype represented an important determinant of outcome. Patients with β^0^/β^0^ genotypes, who completely lack endogenous β-globin production, often showed slower haematological recovery and lower rates of transfusion independence than patients with non-β^0^/β^0^ genotypes. These patients may require higher cell doses and higher vector copy numbers to achieve sufficient therapeutic β-globin expression [53].

The emergence of genome-editing strategies has partly changed this perspective. By targeting the erythroid-specific enhancer of *BCL11A*, CRISPR-Cas-based therapies reactivate endogenous γ-globin expression and increase HbF production. Because this mechanism does not depend on the specific β-globin mutation, it has the potential to overcome genotype-related variability and provide clinical benefit even in patients with severe β^0^/β^0^ genotypes [61]. This mutation-independent mechanism represents one of the main advantages of HbF-reactivating gene editing approaches in TDT and may broaden the population of patients who can benefit from curative autologous gene therapy.

### 5.3. Gene Therapy Versus Allogeneic Haematopoietic Stem Cell Transplantation

Allogeneic haematopoietic stem cell transplantation (allo-HSCT) has historically represented the only established curative treatment for β-haemoglobinopathies [4,10,11]. Its curative potential is based on replacement of the patient’s defective haematopoiesis with donor-derived haematopoietic stem cells capable of producing normal erythroid cells. In patients with an HLA-identical sibling donor, particularly when transplantation is performed at a young age and before the development of advanced organ damage, allo-HSCT can achieve excellent long-term outcomes [10,11]. However, its applicability remains limited by donor availability, the risk of graft rejection, graft-versus-host disease, transplant-related morbidity and mortality, infectious complications, and conditioning-related toxicity [4,10,11].

Autologous gene therapy has emerged as an alternative curative strategy that avoids several immunological limitations of allo-HSCT [12]. Because the patient’s own CD34+ haematopoietic stem and progenitor cells are genetically modified and reinfused, gene therapy eliminates the need for a compatible donor and avoids graft-versus-host disease [12,44]. This is a major conceptual advantage, especially for patients who lack an HLA-matched sibling donor. However, gene therapy does not eliminate the need for intensive treatment: current approved approaches still require stem cell mobilisation, apheresis, ex vivo manufacturing, myeloablative conditioning, prolonged monitoring, and highly specialised centres [28,44].

The choice between allo-HSCT and gene therapy should therefore be individualized rather than viewed as a simple replacement of one strategy by the other. Allo-HSCT remains an important curative option for selected patients, particularly children with severe disease who have an HLA-identical sibling donor and limited organ damage [10,11]. By contrast, gene therapy may be especially attractive for patients without a suitable donor, those at high risk of graft-versus-host disease, or those for whom an autologous approach is preferred [28,44]. Nevertheless, gene therapy introduces specific risks that differ from allo-HSCT, including insertional mutagenesis for integrating lentiviral vectors, off-target or unintended genomic effects for editing platforms, clonal haematopoiesis, complex manufacturing failure, and uncertain long-term durability [21,25,26,27,44].

Both approaches also share important limitations. Myeloablative conditioning, commonly busulfan-based, remains a central source of acute and late toxicity in both allo-HSCT and current gene therapy protocols [44,60]. This includes mucositis, cytopenias, infection risk, hepatic toxicity, gonadal damage, infertility, and possible long-term endocrine or organ complications [44,58,60]. Fertility preservation and careful pre-treatment counselling are therefore essential before either procedure, particularly in children, adolescents, and young adults [44,58].

At present, direct comparative evidence between gene therapy and allo-HSCT is limited. Most gene therapy studies are nonrandomized, open-label, single-arm trials with relatively small cohorts and limited follow-up, whereas allo-HSCT evidence includes longer clinical experience but heterogeneous transplant protocols, donor types, conditioning regimens, and patient populations [26,27,43,47,48]. Consequently, comparative conclusions regarding overall survival, event-free survival, long-term organ protection, fertility outcomes, quality of life, late malignancy risk, and cost-effectiveness remain uncertain [44,47,48]. Future studies and real-world registries should evaluate gene therapy and allo-HSCT within shared outcome frameworks to better define which patients benefit most from each curative strategy.

Overall, allo-HSCT and gene therapy should be considered complementary curative options. Allo-HSCT remains the most established approach when an optimal donor is available and transplant risk is acceptable, whereas gene therapy expands curative potential to patients lacking suitable donors and avoids graft-versus-host disease [10,11,12,28,44]. However, broader use of gene therapy will depend on longer follow-up, safer conditioning regimens, durable efficacy, scalable manufacturing, and equitable access [44,47,48].

### 5.4. Level of Evidence, Long-Term Safety, and Barriers to Broad Implementation

Although gene therapy for β-haemoglobinopathies has rapidly progressed from experimental development to clinical use [62,63], the strength of the available evidence should be interpreted with caution. The most robust clinical data currently come from prospective clinical trials and regulatory approval studies of lentiviral gene addition and CRISPR-Cas9-based gene editing products, which have shown high rates of transfusion independence in TDT and marked reduction or elimination of VOCs in SCD. These results support the clinical validity of approved products in carefully selected patients and represent a major advance over conventional supportive care. However, most available studies are nonrandomized, open-label, single-arm trials, often conducted in relatively small cohorts, with limited follow-up and highly selected eligibility criteria. Therefore, direct comparisons between different products, or between gene therapy and allogeneic HSCT, remain limited. Conclusions regarding comparative effectiveness, long-term organ protection, durability of benefit, late toxicity, and overall survival require further confirmation through longer follow-up, real-world studies, and harmonised registries.

It is also important to distinguish established clinical evidence from early or emerging approaches. Gene addition using lentiviral vectors and gene editing targeting the erythroid-specific enhancer of *BCL11A* currently have the strongest clinical and regulatory support, particularly for selected adolescents and adults with severe disease. By contrast, newer strategies, including base editing, direct correction of disease-causing mutations, alternative nuclease systems, and in vivo delivery platforms, remain largely experimental or in early-phase clinical or preclinical development. These approaches are promising because they may improve precision, reduce genotoxicity, or simplify treatment delivery, but their safety, efficacy, scalability, and long-term durability have not yet been established.

Long-term safety represents one of the most important unresolved issues. Patients treated with gene therapy are expected to live for decades after exposure to genetically modified autologous haematopoietic stem and progenitor cells, myeloablative conditioning, and profound haematopoietic regeneration. Safety concerns differ according to the therapeutic platform. For lentiviral gene addition, the main concern is insertional oncogenesis, in which vector integration near proto-oncogenes or regulatory regions could contribute to clonal expansion or malignant transformation. Although modern self-inactivating lentiviral vectors are considered safer than earlier γ-retroviral vectors, the risk cannot be considered absent. This is reflected by the boxed warning for haematologic malignancy included in the prescribing information for lovotibeglogene autotemcel, which recommends lifelong monitoring and evaluation for haematologic malignancy when clinically indicated.

For genome-editing approaches, including CRISPR-Cas9-mediated disruption of the erythroid-specific *BCL11A* enhancer, the long-term risks are different but equally relevant. Potential concerns include off-target editing, unintended on-target effects, large deletions, chromosomal rearrangements, clonal selection, and delayed haematologic malignancy. Current clinical data have not demonstrated a clear excess of secondary malignancies attributable to approved genome-editing products, but available cohorts remain small, and follow-up is still limited. Therefore, the absence of a detected signal should not be interpreted as definitive evidence of absence of risk. In addition, patients with sickle cell disease may already have increased haematopoietic stress related to chronic inflammation, haemolysis, marrow hyperplasia, transfusion exposure, and repeated regenerative pressure, which may complicate attribution of later clonal or malignant events to the underlying disease, prior therapies, conditioning, or the gene therapy product itself.

Myeloablative conditioning is another major contributor to both early and late toxicity. Busulfan-based conditioning is associated with cytopenias, mucositis, infection risk, hepatic sinusoidal obstruction syndrome, gonadal toxicity, and infertility. In children and adolescents, additional concerns include impaired growth, pubertal delay, endocrine dysfunction, and long-term effects on fertility and quality of life. Therefore, long-term safety assessment should extend beyond malignancy surveillance and include fertility, endocrine function, growth, immune reconstitution, organ function, iron burden, pain outcomes, health-related quality of life, and durability of therapeutic haemoglobin expression or HbF induction.

Current regulatory guidance supports prolonged, risk-based follow-up after gene therapy to detect delayed adverse events. The FDA guidance on long-term follow-up after human gene therapy products recommends structured observation for delayed risks after gene therapy, with follow-up duration adapted to product characteristics, patient-related factors, and preclinical or clinical safety findings. Similarly, EMA guidance emphasizes active clinical follow-up to detect early or delayed adverse reactions, prevent clinical consequences, ensure timely treatment, and collect long-term safety and efficacy information. In practice, post-treatment surveillance should include regular clinical assessment, complete blood counts with differential, monitoring of haemoglobin fractions and therapeutic globin or HbF expression, evaluation of organ function, and assessment of clonal haematopoiesis or vector integration-site patterns when relevant. Bone marrow evaluation should be considered in the presence of unexplained cytopenias, dysplasia, rising blasts, persistent clonal expansion, or other findings suggestive of haematologic malignancy.

Several practical barriers also limit broad implementation. First, current protocols require specialised centres with expertise in haemoglobinopathies, stem cell mobilisation, apheresis, cellular therapy, myeloablative conditioning, and long-term follow-up. Second, manufacturing is complex, costly, and time-consuming, requiring successful collection of adequate CD34+ cells, ex vivo manipulation, quality-control testing, cryopreservation, and coordination between treatment centres and manufacturing facilities. Third, patient selection remains challenging, particularly in children, individuals with advanced organ damage, patients with poor stem cell mobilisation, and those with comorbidities that increase conditioning-related risk. Finally, access and equity remain major unresolved issues, as the regions with the highest burden of sickle cell disease and transfusion-dependent β-thalassaemia often have limited access to specialised centres, long-term surveillance infrastructure, and the financial resources required for these therapies.

Taken together, current evidence supports gene therapy as a transformative and potentially curative option for selected patients with β-haemoglobinopathies, but not yet as a universally applicable intervention. Broader adoption will require longer follow-up, real-world outcome data, clearer comparative studies, safer and less gonadotoxic conditioning regimens, standardised eligibility criteria, scalable manufacturing, harmonised long-term surveillance protocols, and strategies to ensure equitable access in high-prevalence regions.

## 6. Conclusions

Haemoglobinopathies represent a major and growing global public health challenge because of their high prevalence, lifelong morbidity, and substantial impact on survival and quality of life. Over recent decades, advances in the understanding of haemoglobin structure, erythropoiesis, and haemoglobin switching have supported the development of conventional therapies, including chronic transfusion programmes, iron chelation, and pharmacological induction of HbF [4]. More recently, progress in gene transfer and genome-editing technologies has profoundly transformed the therapeutic landscape of β-haemoglobinopathies, offering realistic prospects for curative treatment in both sickle cell disease and transfusion-dependent β-thalassaemia.

Current gene therapy strategies are based mainly on two complementary approaches. Gene addition uses lentiviral vectors to introduce functional or modified β-globin genes into autologous haematopoietic stem and progenitor cells, whereas gene editing modifies endogenous regulatory pathways, particularly those involved in HbF repression, or directly targets disease-causing mutations. Both strategies are designed to restore effective erythropoiesis, reduce disease-related complications, and provide durable clinical benefit.

Clinical studies available to date have shown highly encouraging results. In TDT, gene therapy has enabled many patients to achieve transfusion independence or a substantial reduction in transfusion requirements. In SCD, both gene addition and gene editing have been associated with a marked reduction or complete elimination of severe VOCs. The use of autologous haematopoietic stem cells also avoids several limitations of allogeneic transplantation, including donor availability, graft rejection, and graft-versus-host disease.

Despite these advances, important challenges remain. Current approaches still require myeloablative conditioning, which is associated with acute toxicity, infertility risk, infectious complications, and potential long-term sequelae. Long-term follow-up is also essential to evaluate the durability of response, stability of gene expression or genome editing, clonal haematopoiesis, oncogenic safety, and late adverse events. In addition, the high cost, complex manufacturing process, need for specialised centres, and limited availability in regions with the highest disease burden remain major barriers to equitable access.

Further studies are needed to optimise patient selection, improve conditioning regimens, reduce procedural risks, and clarify long-term outcomes in both adult and paediatric populations. Particular attention should be given to younger patients, in whom early intervention could prevent irreversible organ damage, and to low- and middle-income countries, where haemoglobinopathies are most prevalent. Overall, gene therapy represents one of the most promising frontiers in translational medicine for β-haemoglobinopathies and has the potential to redefine the standard of care by offering a personalised, disease-modifying, and potentially definitive therapeutic approach.

## Figures and Tables

**Table 1:** Main clinical outcomes used to assess the efficacy of gene therapy in sickle cell disease and transfusion-dependent β-thalassaemia.

SCD	TDT
Freedom from vaso-occlusive crises for at least 12 months	Independence from transfusion regimen for at least 22 months
Increased total and fetal Hb levels	Early and clinically significant increase in both total and fetal Hb levels
Improvement in all markers of hemolysis (LDH, haptoglobin)	Improvement of markers of iron overload (reduction of serum ferritin levels and hepatic iron content)
Improvement in both general well-being and overall quality of life	Suspension of iron chelators
	Improvements in erythropoiesis biomarkers
	Improvement in both general well-being and overall quality of life

Adapted from references [26,27]. Abbreviations: Hb, haemoglobin; HbF, fetal haemoglobin; LDH, lactate dehydrogenase; QoL, quality of life; SCD, sickle cell disease; TDT, transfusion-dependent β-thalassaemia; VOCs, vaso-occlusive crises.

**Table 2:** Comparison of approved gene therapy products for sickle cell disease: exa-cel and lovo-cel.

Feature	Exa-Cel	Lovo-Cel
Full name	Exagamglogene autotemcel	Lovotibeglogene autotemcel
Therapeutic strategy	Gene editing	Gene addition
Mechanism of action	Autologous CD34+ haematopoietic stem and progenitor cells are edited ex vivo using CRISPR-Cas9 to disrupt the erythroid-specific enhancer of BCL11A, thereby reactivating endogenous fetal haemoglobin production.	Autologous CD34+ haematopoietic stem and progenitor cells are transduced ex vivo with a lentiviral vector carrying a modified beta-globin gene, leading to production of the anti-sickling haemoglobin HbA^T87Q.
Biological objective	Increase HbF levels to reduce intracellular HbS concentration, inhibit HbS polymerisation, and prevent erythrocyte sickling.	Produce anti-sickling beta-globin to reduce HbS polymerisation, haemolysis, and vaso-occlusive complications.
Current indication discussed in this review	Patients with sickle cell disease aged ≥12 years with recurrent vaso-occlusive crises.	Patients with sickle cell disease aged ≥12 years with recurrent vaso-occlusive crises.
Conditioning regimen	Requires myeloablative conditioning, most commonly busulfan-based.	Requires myeloablative conditioning, most commonly busulfan-based.
Stem cell collection target	Higher CD34+ collection target; approximately 20 × 10^6^ CD34+ cells/kg according to the FDA-label framework cited in the review.	Lower CD34+ collection target than exa-cel; approximately 16.5 × 10^6^ CD34+ cells/kg according to the FDA-label framework cited in the review.
Key efficacy endpoints	Sustained freedom from severe vaso-occlusive crises, together with increased total haemoglobin and HbF levels.	Sustained freedom from vaso-occlusive crises, together with increased total haemoglobin and production of HbA^T87Q.
Main clinical efficacy data	In reported studies, approximately 97% of evaluable patients remained free from vaso-occlusive crises over the assessment period described.	In reported studies, approximately 93% of evaluable patients achieved complete resolution of vaso-occlusive crises for at least 12 consecutive months.
Paediatric/adolescent data	All evaluable paediatric participants treated with exa-cel achieved at least 12 consecutive months without vaso-occlusive crises; improvements in health-related quality of life have also been reported in adolescents.	In the lovo-cel programme, all evaluable paediatric participants treated with the current manufacturing process achieved complete resolution of vaso-occlusive crises during 6–18 months of follow-up.
Safety concerns	Toxicities are largely related to myeloablative conditioning and include cytopenias, infection risk, mucositis, febrile neutropenia, hepatic toxicity, and potential gonadal impairment; additional concerns include unintended or off-target editing effects and long-term clonal safety.	Toxicities are largely related to myeloablative conditioning and include cytopenias, infection risk, mucositis, febrile neutropenia, hepatic toxicity, and potential gonadal impairment; additional concerns include insertional mutagenesis risk and long-term clonal safety because the product uses an integrating lentiviral vector.
Main limitations	Long-term durability, late safety, fertility outcomes, organ protection, and real-world effectiveness require further study. Higher cell collection requirements may be relevant for paediatric patients or poor mobilisers.	Long-term durability, late safety, fertility outcomes, organ protection, and real-world effectiveness require further study. Integrating vector-related risks requires prolonged surveillance.
Comparability between products	Direct comparison with lovo-cel is limited because available studies differ in design, eligibility criteria, baseline patient characteristics, endpoints, manufacturing processes, cell collection targets, and follow-up duration.	Direct comparison with exa-cel is limited because available studies differ in design, eligibility criteria, baseline patient characteristics, endpoints, manufacturing processes, cell collection targets, and follow-up duration.

Adapted from references [26,43,47,48]. Data should be interpreted as an indirect comparison only. No head-to-head randomized trials comparing exa-cel and lovo-cel are currently available, and differences in trial populations, eligibility criteria, product manufacture, cell dose requirements, endpoints, and duration of follow-up limit conclusions regarding comparative efficacy or safety. Abbreviations: CRISPR-Cas9, clustered regularly interspaced short palindromic repeats–CRISPR-associated protein 9; exa-cel, exagamglogene autotemcel; FDA, U.S. Food and Drug Administration; HbA^T87Q, anti-sickling adult haemoglobin containing the T87Q β-globin substitution; HbF, fetal haemoglobin; HbS, sickle haemoglobin; HSPCs, haematopoietic stem and progenitor cells; lovo-cel, lovotibeglogene autotemcel; SCD, sickle cell disease; VOCs, vaso-occlusive crises.

**Table 3:** Ongoing gene therapy clinical trials for sickle cell disease including paediatric or adolescent participants.

	Clinical Trial	Study Population	Mechanism of Gene Therapy (Drug)	Study Design	Primary Efficacy Assessment
Gene Addition	NCT07432867	Severe Sickle Cell Anemia (genotype β^S^β^S^) Age 12–35 years old	Autologous CD34+ Cells Transduced ex vivo by the Bifunctional βAS3m/miR7m Lentiviral Vector Expressing the Therapeutical Beta-globin βAS3m and a Micro-RNA (miRNA) Targeting Specifically the Endogenous βS-globin mRNA (DREAM01)	Phase 1/2 Open Label Cohort Study Single IV infusion Monocentric (France) Time: 24 months after DREAM01 i.v. infusion	Absence of VOCs Platelet and neutrophil recovery Adverse Events Mortality (Transplant-related and All-cause)
	NCT06399107	Severe Sickle Cell Disease (genotypes β^S^β^S^, β^S^β^+^, β^S^β^0^) Age 2–50 years old	Autologous CD34+ HSCs transduced with BAH243 Lentiviral Vector carrying the βA-T87Q-globin gene (BAH243)	Phase 1/2 Open Label Cohort Study Single IV infusion Monocentric (China) Time: 24 months after drug i.v. infusion (then, 13 years follow-up)	Absence of VOCs Globin Response
Gene Editing	NCT06647979	Severe Sickle Cell Disease (genotypes β^S^β^S^, β^S^β^0^, β^S^β^D^, β^S^β^O^) Age 13–40 years old	Autologous bone-marrow-derived CD34+ HSPCs electroporated with BCL11A-enhancer-targeting Cas9 ribonucleoprotein (No drug name)	Phase 1/2 Open Label Cohort Study Single IV infusion Monocentric (USA) Time: 24 months after drug i.v. infusion	Absence of severe VOCs Primary Engraftment Adverse Events Mortality
	NCT04819841	Severe Sickle Cell Disease Age 12–40 years old	Autologous CRISPR-Cas9 edited and sickle mutation-corrected HSPC to Convert HbS to HbA (NULA-CEL)	Phase 1/2 Open Label Cohort Study Single IV infusion Multicentric (USA) Time: 24 months after NULA-CEL i.v. infusion	Absence of severe VOCs Neutrophil recovery Adverse Events Treatment-related Mortality Overall Survival

Adapted from reference [50]. Abbreviations: Cas, CRISPR-associated protein; CD34+, cluster of differentiation 34-positive haematopoietic stem/progenitor cells; CRISPR, clustered regularly interspaced short palindromic repeats; Hb, haemoglobin; HSCs, haematopoietic stem cells; HSPCs, haematopoietic stem and progenitor cells; i.v., intravenous; miRNA, microRNA; VOCs, vaso-occlusive crises.

**Table 4:** Proposed eligibility criteria for gene therapy in patients with sickle cell disease.

Present Regulatory Agency Criteria	Potential Future Candidates	Not Eligible Patients
Patients followed by a comprehensive center for hemoglobinopathies Genotype β^S^β^S^, β^S^β^+^ and severe β^S^β^0^ No HLA-matched family donor Patients over 12 years of age 2 VOC requiring hospitalization per year in the 2 previous years with no response to HC at MTD, either alone or in combination with other treatments Having at least 2 ACS in the prior 2 years	Patients followed by a comprehensive center for hemoglobinopathies Genotype β^S^β^S^, β^S^β^+^ and severe β^S^β^0^ No HLA-matched family donor Patients aged more than 2 years and <45 years at least 2 hospitalized VOCs per year in the 2 previous years with no response to HC at the MTD, either alone or in combination with other treatments Recurrence of ACS in spite of HC at MTD Diastolic dysfunction in the absence of restrictive myocardiopathy PH defined as a mean pulmonary arterial pressure between 25 and 29 mmHg defined by heart cardiac catheterization Chronic cholangiopathy/hepatopathy without hepatic failure Chronic kidney disease ≤2 stage with or without ACE or ARB treatment Urine albumin/creatinine ratio >30 mg/mmol without renal failure Persistently abnormal TCD velocities despite HU at MTD Significant cerebrovascular disease treated with regular blood transfusions History of HTR/severe hemolytic reaction? Co-existent auto-immune disease	Baseline HbF > 30% Organ dysfunction not compatible with myeloablative conditioning regimen Active infection (HBV, HCV, HIV) Patients with NYHA III or above PH with pulmonary arterial pressure >30 mmHg defined by heart cardiac catheterization Significant arrhythmia requiring therapy Myocardial ischemia in the previous 12 months Restrictive myocardiopathy Chronic HBV and HCV infection Liver fibrosis grade ≥3 Liver cirrhosis If LIC >7 mg/Fe/gr liver, iron chelation therapy should be started until LIC <7 mg/Fe/gr liver CKD stage 3−4 or higher End-stage renal disease (ESRD) under hemodialysis Severe cerebrovascular disease with moyamoya Lupus anticoagulant (LAC) or anti-phospholipids

Adapted from references [28,41]. Abbreviations: ACE, angiotensin-converting enzyme; ACS, acute chest syndrome; ARB, angiotensin receptor blocker; CKD, chronic kidney disease; EHA SWG, European Hematology Association Specialized Working Group; EBMT, European Society for Blood and Marrow Transplantation; HbF, fetal haemoglobin; HBV, hepatitis B virus; HCV, hepatitis C virus; HIV, human immunodeficiency virus; HLA, human leukocyte antigen; HTR, haemolytic transfusion reaction; LIC, liver iron concentration; MTD, maximum tolerated dose; NYHA, New York Heart Association; PH, pulmonary hypertension; SCD, sickle cell disease; TCD, transcranial Doppler; VOC, vaso-occlusive crisis.

## Data Availability

No new data were created or analyzed in this study. Data sharing is not applicable to this article.
